# Situation of Self-Reported Anxiety and Depression among Urban Refugees and Asylum Seekers in Thailand, 2019

**DOI:** 10.3390/ijerph18147269

**Published:** 2021-07-07

**Authors:** Nareerut Pudpong, Hathairat Kosiyaporn, Mathudara Phaiyarom, Watinee Kunpeuk, Pigunkaew Sinam, Sataporn Julchoo, Rapeepong Suphanchaimat

**Affiliations:** 1 International Health Policy Program, Ministry of Public Health, Nonthaburi 11000, Thailand; hathairat@ihpp.thaigov.net (H.K.); mathudara@ihpp.thaigov.net (M.P.); watinee@ihpp.thaigov.net (W.K.); pigunkaew@ihpp.thaigov.net (P.S.); sataporn@ihpp.thaigov.net (S.J.); rapeepong@ihpp.thaigov.net (R.S.); 2 Sirindron College of Public Health, Chonburi 20000, Thailand; 3 Division of Epidemiology, Department of Disease Control, Nonthaburi 11000, Thailand

**Keywords:** mental health, anxiety, depression, urban, refugees, asylum seekers

## Abstract

Academic evidence on the health of urban refugees and asylum seekers (URAS) in Thailand is extremely sparse, especially for neglected problems such as mental health disorders. This study aimed to investigate the prevalence of anxiety and depression and factors associated with these problems among URAS in Bangkok. A cross-sectional study was conducted in 2019. The URAS were randomly selected from the roster of the Bangkok Refugee Centre (BRC). A self-administered questionnaire was used and 180 samples were recruited. Descriptive statistics and multivariable logistic regression were used for the analysis. We found a prevalence of 70.0% for anxiety and 39.5% for depression. Compared to Southeast Asia and China, URAS from other regions were 3.4 times (95% CI 1.5–7.5, *p* < 0.05) and 4.0 times (95% CI 1.1–14.0, *p* < 0.05) more likely to experience anxiety and depression, respectively. URAS with chronic co-morbidities (OR = 3.4, 95% CI 1.2–9.4, *p* < 0.05) and being divorced or widowed (OR = 11.1, 95% CI 2.1–57.2, *p* < 0.05) faced greater odds of depression than those without co-morbidities and being single. Related health authorities should play a proactive role in providing mental healthcare services for URAS, with greater consideration for those of certain nationalities and with chronic diseases.

## 1. Introduction

According to the United Nations High Commissioner for Refugees (UNHCR), in 2018 there were over 70.8 million forcibly displaced people worldwide [1]. Of these people, 25.9 million were refugees and asylum seekers (RAS) [2]. The majority of RAS reside in overcrowded reception facilities. Some are captive in long-term detention centres and experience relatively poor mental health [3,4]. Many of them end up living in intermediate countries while awaiting approval to seek refuge in their proposed destinations.

Mental health is one of the most neglected health problems in RAS. Evidence suggested that a number of RAS experience a variety of mental health disorders, including anxiety and depression [5,6], and these problems ultimately lead to a compromise in their overall well-being [7]. The prevalence of anxiety and depression in RAS varies from study to study. In Germany, many asylum seekers, identified through health screening, were found to have mental health problems, with 40.7% experiencing anxiety disorders and 54.2% experiencing depression [8]. An umbrella review by Turrini et al. suggested the prevalence of anxiety (4–40%) and depression (5–44%) in about 40.0% of RAS [9]. A systematic review and meta-analysis study conducted across 15 countries found that the prevalence of depression and anxiety in RAS reached up to 31.5% and 11.0%, respectively [10]. A study in Sweden revealed significant levels of mental illness among Syrian refugees, including 31.8% experiencing anxiety and 40.2% experiencing depression [11].

Regarding predictive factors of mental illness, a study among RAS in Ireland observed a strong relationship between loneliness and communication problems, and depression [12]. Similarly, the high prevalence of depression (36.1%) among African refugees in Hong Kong was associated with being alone (lack of support from families and friends), self-reported poor health, and experiencing discrimination [13]. Tinghög et al. found that these mental health problems were significantly associated with being women, older, and divorced/widowed [11]. Another study in Germany found that lack of information from families and perceived health need among recently arrived refugees showed a strong link with depression, somatization and anxiety [14].

Previous literature has suggested that social determinants influencing RAS mental health include income, employment, language difference, the asylum seeker process, social support and isolation, and discrimination [15]. For instance, Colombian refugees in Ecuador who experienced discrimination and social isolation had high levels of stress, anxiety, and depression [16]. For refugees in Canada, the struggle to find work and being overqualified for employment resulted in self-reported poor mental health [17]. Unemployment can also affect not only RAS economic well-being, but also their social status and sense of self-esteem [18]. Policies to address the mental health of RAS need to focus on social inclusion. For example, in Australia and New Zealand, mental health screening on arrival, access to primary care and mental health services, and language support have been provided for RAS [19]. In European countries, social integration measures, such as education, employment, cultural mediators, and language interpreters have been established to assist RAS settlement [20]. Refugee-friendly services have also been provided in European countries to ensure access to quality health care [21,22].

Thailand plays a vital role in international migration in Southeast Asia. It is one of the most common destination sites for human migration, including refugees, asylum seekers, displaced persons, migrant workers and even foreign professionals (numbering almost five million in total) [23]. This is partly due to the country’s geographical advantage that aligns it in the centre of the Southeast Asia Peninsula and the leap-and-bound economic growth in the last decade. In terms of health support for migrants, Thailand has been recognized for its success in protecting the health of migrant workers, particularly those from Cambodia, Lao PDR, Myanmar and Vietnam (CLMV nations). One of the most renowned achievements is the advancement of public health insurance to cover both documented and undocumented CLMV workers. Undocumented migrant workers are eligible for public health insurance (namely, the Health Insurance Card Scheme (HICS)) managed by the Ministry of Public Health (MOPH) once they register with the Ministry of Interior (MOI) to acquire legitimate residence permit, and with the Ministry of Labour (MOL) for work-permit acquisition [24]. The HICS benefit covers a vast range of health care, from basic outpatient care to high-cost admission [25]. 

Despite the remarkable progress in providing health protection for migrant workers, RAS seem to be neglected on the political radar [26]. Thailand neither takes part in the 1951 Refugee Convention nor has any specific legal framework for RAS protection [27]. Moreover, even among RAS, the policy ‘intensity’ in terms of health protection is diverse. Refugees in sheltered areas along the Thai border have been recognized by the Thai government for years (since the exodus of people from Myanmar into Thailand in 1988; n~100,000). The health of these sheltered refugees is de facto covered by numerous charitable agencies in the shelters, such as the American Refugee Committee International, as well as nearby district hospitals if the patients’ disease conditions are beyond the care capacity of these agencies [28]. In contrast, RAS in urban settings (so-called urban refugees and asylum seekers (URAS)) appear to be overlooked by the policy [29]. Currently, more than 5000 URAS live in Thailand, having migrated from numerous countries (such as China, Pakistan, and Vietnam). Almost all of them reside in Bangkok without lawful residence permits. Some entered the country lawfully from the outset but later became over-stayers [29].

So far, academic evidence on the health of URAS in Thailand is extremely sparse, especially for neglected health problems, such as mental health disorders. Therefore, the objectives of this study were to explore the magnitude of mental health problems among URAS in Thailand and identify related factors that may link to their mental health problems. 

## 2. Conceptual Framework 

**Figure 1:**
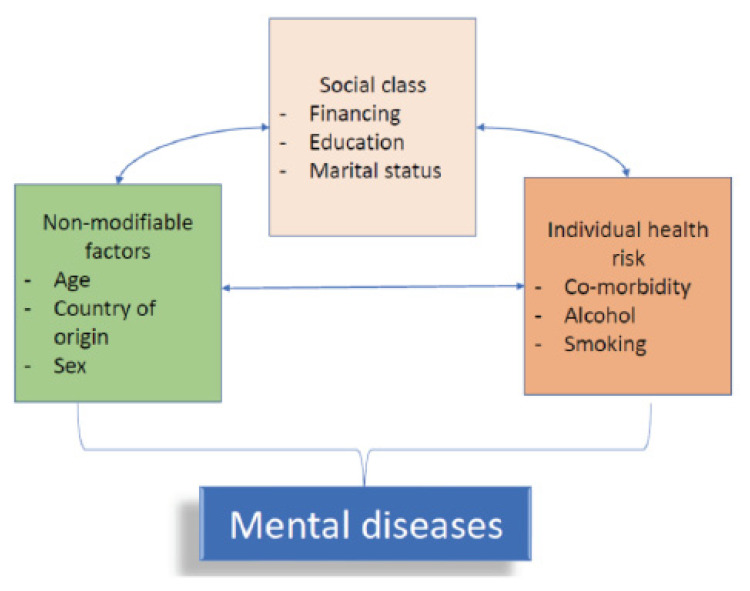
Conceptual framework for the analysis.

The conceptual framework of this study was developed using the social determinants of health (SDH) proposed by the World Health Organization in 2010 [30,31], Figure 1.

The framework suggests that the well-being of an individual is influenced by numerous social determinants. These determinants include non-modifiable factors (such as sex, country of origin, and age), factors that relate to social class (such as financing, education and marital backgrounds) and individual health risks (such as co-morbidity, alcohol drinking, and smoking history). All these determinants were considered in the analysis. We hypothesized that these factors might influence each another, thus warranting the use of multivariable analysis for simultaneous adjustment.

## 3. Methods

### Study Design and Participants

A cross-sectional study was conducted between October and December 2019 [32]. We collaborated with the Bangkok Refugee Centre (BRC), the main civic group under the patronage of UNHCR, to select participants from BRC’s URAS roster. We focused on the top ten nationalities of URAS, which included Afghan, Cambodian, Chinese, Iraqi, Pakistani, Palestinian, Somali, Sri Lankan, Syrian, and Vietnamese, totalling 3021 participants. 

A sample size was calculated with the aim of quantifying the prevalence of anxiety and depression. Therefore, the formula was—*n* = z^2^p (1 − p)/d^2^ where z = 1.96 (reflecting z-statistic for two-tailed 95% confidence level), p reflects the prevalence of anxiety disorder, and d denotes acceptable error. As, so far, there has been no prior research on anxiety prevalence in URAS in Thailand, we replaced ‘p’ with 0.103, which is anxiety prevalence (10.26%) among asylum seekers in Switzerland as reported by Maier et al. [33]. We also replaced ‘d’ with 0.05. After applying all parameters in the formula, 142 samples were needed. When accounting for a 20% non-response rate and incomplete information, the final sample volume reached 170. We used stratified random sampling with probability proportional to size (PPS) to account for the distribution of age, sex and nationality. Supplementary File S1 shows the list of samples in the sex, age and nationality strata combined. 

In the actual survey, BRC staff were able to randomly recruit 206 participants; slightly larger than the calculated samples. However, 23 people refused to participate in the survey, and 3 people did not provide adequate demographic information in relation to the study framework. Therefore, these participants were excluded, making the final total of 180 samples in the survey.

## 4. Data Collection

The data collection began with a preparation meeting among BRC coordinators and a research team to finetune understandings on the questionnaire and survey methods. Then, the BRC volunteer asked the selected URAS to visit the BRC office. Each participant was asked to complete the paper questionnaire by him/herself but the BRC staff were always available to assist if a participant did not understand the questionnaire. For illiterate participants, a verbal interview was used. The questionnaire was translated into various languages based on URAS nationalities. Each respondent took about 30 min to complete the questionnaire. The data collection was conducted after receiving approval from the Institute of the Development of Human Research Protections (IHRP), Thailand (letter head—IHRP 595/2562). Written consent and a participant information sheet were distributed to all participants before starting the fieldwork. All participants received a stipend of about US $10 to subsidize travel expenses. 

### 4.1. Measures

The measurement tool was the questionnaire applied from the annual Thai Health Welfare Survey, which was composed of two parts—(i) demographic characteristics; and (ii) mental health questions focusing on anxiety and depression. We divided independent variables into three main groups corresponding to our conceptual framework, including—(1) non-modifiable factors (gender, age, religion, region, and period of living in Thailand); (2) social class factors (education, marital status, and financial status); and (3) individual risk factors (chronic disease, alcohol drinking, and smoking).

Two dependent variables were considered in this study, which were anxiety and depression. For anxiety, we asked “Currently, regarding your anxiety, what do you think about it most?” The answer was arranged in 5 scales—none, low, medium, high, and very high. In the analysis, for communication convenience, we re-categorized the answer into two groups—No anxiety (none) versus Having anxiety (low to very high). For depression, we used the Depression Assessment Question of the Department of Mental Health of the MOPH. The question comprised nine items, asking about their feelings that showed signs of depression in the past 2 weeks, with four scoring levels—no = 0, sometimes (1–<7 days) = 1, often (≥7 days), and everyday = 3 (possible highest score was 27). Details of each question item are presented in Supplementary File S2.

We grouped the depression score into two groups—No depression (<7) versus Having depression (≥7). Regarding the country-of-origin variable, we divided it into two groups—(i) Southeast Asia and China and (ii) others. This is because we assumed that participants from Southeast Asia and China were more familiar with the culture in Thailand than other participants.

### 4.2. Analysis

We started by describing the data with descriptive statistics, using number and frequencies. Then we used univariable analysis to explore associations between mental health problems (anxiety and depression) and each variable. In this step, the significance level from univariable analysis was evaluated against the *p*-value of lower than 0.05. Variables showing statistical significance in univariable analysis would be included in the multivariable analysis. For a group variable that contained three or more levels, Chi-square or Fisher’s exact test was applied to obtain the overall *p*-value. Note that Fisher’s exact test was employed if expected values in each cell from the cross-tabulation was lower than five. We then employed multivariable logistic regression to account for independent variables all at once. Odds ratios (OR) and 95% confidence intervals (95% CI) were reported. All analyses were performed by STATA V.13.1 (serial number: 401406358220). An inverse probability weighting was applied to account for the sampling design.

### 4.3. Results

The key characteristics of URAS are presented in Table 1. Males slightly outnumbered females. Most of them were in the working age group (53.3%), single (52.8%) and had completed primary education (66.1%). Almost half of the participants had monthly income lower than their overall expenses (41.7%), and had been living in Thailand for at least five years (42.8%). About one-third of them came from Southeast Asia and China (40.0%), while the rest were from Middle East, South Asia, and Africa (60.0%). Almost half of them were Muslim (47.2%), followed by Christians (40.6%). The majority of them did not have chronic diseases (77.2%). Most of them neither drank alcohol nor smoked (93.9% and 91.1%, respectively). 

Table 2 presents the prevalence self-reported anxiety and depression among URAS in Thailand. Approximately two-thirds of URAS reported experiencing anxiety (70.0%), whereas just above one-third of them experienced depression (39.5%).

Table 3 shows the results of multivariate regression analyses on anxiety. The crude analysis on anxiety found that URAS with higher education (degree or above) had greater odds of anxiety than those with primary school education (*p*-value < 0.001). In addition, URAS in the working age group and those originating from outside Southeast Asia and China were more likely to feel anxious than others. Being married and having chronic diseases appeared to have a positive relationship with anxiety. After adjusting for all significant variables from univariate analysis, URAS from outside Southeast Asia and China were about three times more likely to experience anxiety than those migrating from these areas (adjusted OR = 3.4; 95% CI = 1.5–7.5; *p*-value = 0.003). Despite no statistical significance shown in most variables (the significant level was more relaxed at *p*-value = 0.10), education and age still exhibited a significant relationship with anxiety. The odds of anxiety among participants with education achievement were about 11 times greater than those with primary education and the odds of anxiety among adults (15–60 years) were about three times greater than children.

The results of multivariate regression analyses on depression are shown in Table 4. For depression, the univariate estimation showed a statistical significance in the following variables—marital status, region of origin, religion and encountering chronic diseases. In multivariable analysis, participants from outside Southeast Asia and China were about four times more likely to suffer from depression than those from within, with a *p*-value of 0.035. Having chronic disease exhibited a strong significant level with depression (*p*-value = 0.003). In addition, divorced participants or widowers were 11 times more likely to be depressed compared to those who were single (*p*-value = 0.004). If the significance level was more relaxed at 10% confidence level, the odds of depression among Muslim participants were about 10 times significantly greater than Buddhists. The multivariate models exploring the effects of three different independent variable groups on the study outcomes were also exercised (see Supplementary File S3).

## 5. Discussion

Overall, this study suggested a prevalence of 70.0% for anxiety and 39.5% for depression among URAS in Thailand. The prevalence of anxiety among URAS is much higher than in the Thai population, which is only 0.3% [34], whereas the prevalence of depression is slightly lower than in the Thai population, which is about 48.5% [35]. When compared to RAS in other places, the anxiety prevalence was relatively much higher than that reported in previous literature, while the depression prevalence was similar to previous observations. For instance, Turrini et al. conducted umbrella reviews and found that the prevalence of mental health problems among refugees was about 4–40% for anxiety and 5–44% for depression [9]. Similarly, Blackmore et al. undertook a systematic review and meta-analysis study, and revealed a low level of anxiety of only 11.0%, and a level of depression of 31.5% [10]. One of the explanations for the extremely high prevalence of anxiety in this study was the self-perception of each individual towards the question. In this case, we asked only if the participants feel anxious about their daily life, and so the answer was their perceived illness. In contrast, many other studies used a set of different questions to assess anxiety; for instance, the Hopkins Symptom Checklist (HSCL-25) [36,37]. 

On the contrary, we assessed depression by using a set of nine questions from the Mental Health Department, MOPH, to assess participants’ depression during the last two weeks (see Section 3), which was the same as the PHQ-9 assessment used by several previous RAS studies [38].

Regarding factors associated with anxiety, we found that only the non-modifiable factor of region was significantly associated with anxiety among URAS in Thailand (after adjusting for potential confounders). For depression, the study found that three factors in different SDH groups, including marital status (social class factor), region (non-modifiable factor) and chronic diseases (individual risk factor) were strong predictors for depression among URAS in Thailand. 

Additionally, the finding that a social class factor like ‘education’ and a non-modifiable factor like ‘age’ were predictive factors for anxiety was in line with the study among Syrian refugees in Sweden, which found a higher prevalence of anxiety among refugees with university degrees (30.6%) than those without (29.8%), and among older people (41.9%) than younger age groups (26.2–34.2%) [11]. A possible explanation about education is that URAS with higher educational backgrounds might be used to better living conditions and work benefits than those with fewer educational qualifications. When they migrate to Thailand, according to current laws, URAS are not allowed to obtain work permits. Therefore, those with higher educational status might face more severe lifestyle adjustments than those without. Although the prevalence of anxiety was more pronounced in the elderly in prior literature [11], this study found that anxiety problems were more prevalent among working-age adults. This issue may be related to struggling in job finding among URAS adults—as is mentioned above, the Thai law still prohibits the acquisition of work permit in URAS. Nevertheless, this is still an indicative assumption. Further studies on the interaction between job acquisition and mental health among URAS in Thailand are worthwhile. However, the inextricable connection between employment and mental health has been shown in many settings, such as among Iraqi refugees in the US, Southeast Asian refugees in Canada, and African refugees in Australia [39,40,41]. Previous evidence has suggested that unemployment is linked with inadequate standards of living, limited access to proper social healthcare and support, unhealthy behaviours, and mental health disorders; while employment may lead RAS to confront and accommodate language barriers, unfamiliar cultures and the social nuances of the new community [40,41]. Discrimination in the labour market results in stress, anxiety, and depression [41]. Nevertheless, the benefits of employment outweigh the adverse effects, and therefore, providing employment opportunities and promoting social integration for refugees is recommended as a policy option [39,40]. Note that the finding that showed a significant relationship between being widowed or divorced with anxiety aligned with the observation in the previous study [11].

The region or country of origin, outside of Southeast Asia and China, was another non-modifiable factor that exhibited a strong positive relationship with both anxiety and depression in this study. This finding corresponds to a previous study by Fazel et al. which indicated that non-western refugees living in western countries tended to experience depression more than other ethnic groups [5]. This observation could also be explained by the cultural differences across nations. A study by Tribe also suggested that culture is one of many factors that is linked with mental illness among refugees [42]. A study on Syrian refugees in Switzerland also showed that facing multiple structural and social-cultural barriers could hinder access to mental healthcare services [43]. This notion might be a solid explanation for this study’s results, as the URAS originating from Southeast Asian nations and China might be more familiar with the Thai culture than those originating from elsewhere. The same explanation can apply for the finding above where Christians and Muslims are more likely to feel depressed than Buddhist URAS, as over 90% of Thai citizens identify as Buddhists [44]. 

The study found, in both univariable and multivariable analyses, that an individual risk factor, such as having chronic diseases, was significantly associated with depression. This statement concurs with growing evidence that non-communicable diseases have become a new challenge in the health protection of refugees [45]. Generally, there is a linkage between physical and mental health. A study among Syrian refugees in the Netherlands found that refugees usually had at least one chronic condition as well as depression and anxiety symptoms [46]. Another study among African refugees in Hong Kong found a significant relationship between chronic illness and depression in the univariable analysis [13]. In addition, a study among RAS in Ireland revealed a significant relationship between chronic conditions and depression and anxiety symptoms in multivariable analyses [12]. 

All of the above findings reveal some important policy implications. First, the prevalence of anxiety and depression among URAS is extremely high. This alludes to the fact that URAS might face barriers of access to mental healthcare. Second, the findings could help with the prioritisation of policy implementation to support mental healthcare. For example, the provision of care should initially target URAS from regions outside Southeast Asia and China. Moreover, mental healthcare design should seriously consider and account for cultural differences. Developing a refugee-friendly service is worth considering. European countries have been developing refugee-friendly services to ensure access to quality care for vulnerable populations; for example, child-friendly services [21,22].

There remain some limitations of the study. First, anxiety and depression are intertwined with post-traumatic stress disorder (PTSD), which is a common illness among URAS identified in much of the previous literature. However, this study was not designed to explore this issue from the outset and the diagnosis of PTSD needs detailed information obtained through physical examination and a psychiatrist’s interview. Second, the term ‘anxiety’ used in this study was based on self-perception. Thus, it does not necessarily reflect an actual anxiety disorder. Future studies that apply a more standardized tool to assess anxiety disorders, for instance, the Hopkins Symptom Checklist (HSCL-25), are recommended. Third, this present study investigated mental health among those living in urban areas. Therefore, the findings may not represent mental health problems among URAS elsewhere, such as those in sheltered areas or in detention centres. Fourth, the nature of cross-sectional design means that it is difficult to conclude the causal inference between dependent variables (mental problems) and independent variables (participants’ characteristics). To reach a strong conclusion on causal relationships between mental health and associated factors, continuous monitoring of the health of URAS as a whole (including mental health) is required and this will definitely be useful for future planning of healthcare provision for URAS. Fifth, this study has not delved into the root causes of anxiety and depression. These may include experiences of discrimination and racism, and also trust in the healthcare system [12,13,15]. Further qualitative research on these issues is of much value. Lastly, as some URAS refused to participate in the survey and some did not provide demographic data of interest to be used for the analysis, this might somehow reduce the statistical power at a certain level. However, there is generally no big difference in the characteristics of URAS. They aimed to participate in the study from the beginning and most of them finally joined the survey (as displayed in Supplementary File S1). This meant the potential selection bias might not be severe, though we were aware of its existence.

## 6. Conclusions

This study appears to be among the first studies that examines mental health problems, especially anxiety and depression, among URAS in Thailand. The findings reveal that URAS face a high prevalence of anxiety and depression, compared with Thai residents, as indicated in previous literature. The study also confirmed that social determinants, including the non-modifiable factor, social class, and individual risk factors could influence the health and well-being of URAS. In addition, associated factors for the increased likelihood of mental health disorders included having migrated from countries other than Southeast Asia and China, being divorced or widowed and having chronic diseases as co-morbidities. Further studies should employ a more in-depth qualitative approach to explore the root causes and to increase understandings about social dimensions of mental health among URAS. Other social class factors, such as employment, could also affect the mental health of URAS since it is likely related to their financial status, standards of living, and access to health services and social support. Hence, the granting of the right to work for URAS should be considered as an important policy option as it would help them gain increased self-reliance and dignity and improve their mental health. Continuous monitoring on the overall health of URAS, including mental health problems, will be helpful for effective health system design for the future. 

## Figures and Tables

**Table 1:** Characteristics of urban refugees and asylum seekers in Thailand in 2009.

Characteristics	Number of Respondents	Percentage
**Gender**		
Female	88	48.9
Male	92	51.1
**Age (years)**		
<15	75	41.7
15–60	96	53.3
>60	9	5.0
**Region**		
South East Asia and China	72	40.0
Others *	108	60.0
**Religion**		
Buddhism	15	8.3
Christ	73	40.6
Muslim	85	47.2
Others **	7	3.9
**Period of living in Thailand (years)**		
<5	62	34.4
≥5	77	42.8
Not answer	41	22.8
**Education**		
Up to primary level	119	66.1
Up to secondary level	41	22.8
Degree or above	20	11.1
**Marital status**		
Single	95	52.8
Married	79	43.9
Widow/divorced/separated	4	2.2
Not answer	2	1.1
**Financial status**		
Income lower than expense	75	41.7
Income equal to expense	44	24.4
Income higher than expense	24	13.3
Not answer	37	20.6
**Chronic diseases**		
No	139	77.2
Yes	39	21.7
Not answer	2	1.1
**Alcohol drinking**		
No	169	93.9
Yes	7	3.9
Not answer	4	2.2
**Smoking**		
No	164	91.1
Yes	14	7.8
Not answer	2	1.1
Total	180	100

* Other regions e.g., Middle East, South Asia, Africa; ** Other religions e.g., Hindu, Ahmadi Muslim, and Falun Gong.

**Table 2:** Prevalence of anxiety and depression among urban refugees and asylum seekers in Thailand in 2009.

Mental Health Problem	Number of Respondents	Percentage
**Anxiety**		
Not anxious	51	28.3
Anxious	126	70.0
Not answer	3	1.7
**Depression**		
Not depressed	94	52.2
Depressed	71	39.5
Not answer	15	8.3
Total	180	100

**Table 3:** Multiple regression analyses on anxiety among urban refugees and asylum seekers in Thailand in 2019 (*n* = 177).

	Independent Variables	Number of Respondents Who Are Not Anxious (%)	Number of Respondents Who Are Anxious (%)	Crude OR (95% CI)	*p*-Value of Crude OR	Adjusted OR (95% CI)	*p*-Value of Adjusted OR
Non-modifiable factor	**Gender**						
	Female	26 (51.0)	61 (48.4)	1.0			
	Male	25 (49.0)	65 (51.6)	1.1 (0.5–2.2)	0.816		
	**Age (years)**						
	<15	36 (70.6)	37 (29.4)	1.0		1.0	
	15–60	14 (27.4)	81 (64.3)	5.6 (2.6–12.0)	<0.001	3.4 (0.8–13.9)	0.084
	>60	1 (2.0)	8 (6.3)	6.6 (0.7–63.5)	0.102	0.5 (0.02–10.0)	0.637
	**Region**						
	South East Asia and China	29 (56.9)	40 (31.7)	1.0		1.0	
	Others	22 (43.1)	86 (68.3)	2.9 (1.4–5.8)	0.003	3.4 (1.5–7.5)	0.003
	**Religion**						
	Buddhism	7 (13.7)	8 (6.3)	1.0			
	Christ	24 (47.1)	46 (36.5)	1.8 (0.6–5.6)	0.315		
	Muslim	18 (35.3)	67 (53.2)	3.6 (1.1–11.1)	0.029		
	Others	2 (3.9)	5 (4.0)	3.2 (0.5–20.8)	0.225		
	**Period of living in Thailand (years) (*n =* 137)**						
	<5	19 (54.3)	41 (40.2)	1.0			
	≥5	16 (45.7)	61 (59.8)	1.8 (0.8–4.2)	0.157		
Social class factor	**Education**						
	Up to primary level	40 (78.4)	77 (61.1)	1.0		1.0	
	Up to secondary level	10 (19.6)	30 (23.8)	1.5 (0.7–3.3)	0.346	0.5 (0.2–1.4)	0.182
	Degree or above	1 (2.0)	19 (15.1)	43.1 (5.4–343.5)	<0.001	11.2 (0.8–163.4)	0.076
	**Marital status (*n =* 175)**						
	Single	41 (82.0)	53 (42.4)	1.0		1.0	
	Married	8 (16.0)	70 (56.0)	6.3 (2.7–14.5)	<0.001	2.8 (0.8–9.9)	0.117
	Widow/divorced/separated	1 (2.0)	2 (1.6)	2.1 (0.2–19.5)	0.506	0.8 (0.1–6.6)	0.819
	**Financial status (*n =* 143)**						
	Income lower than expense	25 (56.8)	50 (50.5)	1.0			
	Income equal to expense	13 (29.6)	30 (30.3)	1.1 (0.5–2.7)	0.756		
	Income higher than expense	6 (13.6)	19 (19.2)	1.7 (0.5–6.1)	0.398		
Individual risk factor	**Chronic diseases (*n =* 175)**						
	No	46 (92.0)	90 (72.0)	1.0		1.0	
	Yes	4 (8.0)	35 (28.0)	4.8 (1.5–15.1)	0.007	2.5 (0.6–10.4)	0.217
	**Alcohol drinking (*n =* 174)**						
	No	50 (98.0)	117 (95.1)	1.0			
	Yes	1 (2.0)	6 (4.9)	2.3 (0.3–19.8)	0.449		
	**Smoking (*n =* 175)**						
	No	49 (96.1)	113 (91.1)	1.0			
	Yes	2 (3.9)	11 (8.9)	2.3 (0.5–10.8)	0.290		
	**Total**	51	126				

Note: 1. *p*-value for group variables by Fisher’s exact test ≤ 0.001 (age), 0.016 (education), < 0.001 (marital status) and 0.162; (religion). *p*-value for a group variable by Chi-square test = 0.488 (financial status); 2. Variables that did not exhibit significant results in the univariate analysis were not included in the multivariate analysis.

**Table 4:** Multiple regression analyses on depression among urban refugees and asylum seekers in Thailand in 2019 (*n =* 165).

	Independent Variables	Number of Respondents Who Are Not Depressed (%)	Number of Respondents Who Are Depressed (%)	Crude OR (95% CI)	*p*-Value of Crude OR	Adjusted OR (95% CI)	*p*-Value of Adjusted OR
Non-modifiable factor	**Gender**						
	Female	45 (47.9)	39 (54.9)	1.0			
	Male	49 (52.1)	32 (45.1)	0.8 (0.4–1.5)	0.401		
	**Age (years)**						
	<15	46 (48.9)	26 (36.6)	1.0			
	15–60	47 (50.0)	40 (56.3)	1.6 (0.8–3.1)	0.204		
	>60	1 (1.1)	5 (7.1)	8.5 (0.7–96.0)	0.085		
	**Region**						
	South East Asia and China	53 (56.4)	13 (18.3)	1.0		1.0	
	Others	41 (43.6)	58 (81.7)	5.5 (2.6–11.7)	<0.001	4.0 (1.1–14.0)	0.032
	**Religion**						
	Buddhism	14 (14.9)	1 (1.4)	1.0		1.0	
	Christ	45 (47.9)	22 (31.0)	8.0 (1.0–65.3)	0.054	7.6 (0.6–91.9)	0.109
	Muslim	30 (31.9)	46 (64.8)	25.4 (3.1–206.0)	0.002	10.3 (0.7–154.8)	0.091
	Others	5 (5.3)	2 (2.8)	7.8 (0.6–105.6)	0.122	3.9 (0.2–88.2)	0.390
	**Period of living in Thailand (years) (*n =* 125)**						
	<5	31 (49.2)	22 (35.5)	1.0			
	≥5	32 (50.8)	40 (64.5)	1.8 (0.8–3.8)	0.134		
Social class factor	**Education**						
	Up to primary level	66 (70.2)	44 (62.0)	1.0			
	Up to secondary level	24 (25.5)	14 (19.7)	0.8 (0.4–1.8)	0.660		
	Degree or above	4 (4.3)	13 (18.3)	4.7 (1.4–16.0)	0.015		
	**Marital status (*n =* 163)**						
	Single	56 (60.9)	34 (47.9)	1.0		1.0	0.338
	Married	35 (38.0)	34 (47.9)	1.6 (0.8–3.2)	0.167	1.5 (0.7–3.5)	0.004
	Widow/divorced/Separated	1 (1.1)	3 (4.2)	5.4 (0.6–48.1)	0.131	11.1 (2.1–57.2)	0.004
	**Financial status (*n =* 135)**						
	Income lower than expense	37 (50.7)	35 (56.4)	1.0			
	Income equal to expense	19 (26.0)	22 (35.5)	1.2 (0.5–2.7)	0.711		
	Income higher than expense	17 (22.3)	5 (8.1)	0.3 (0.1–1.1)	0.071		
Individual risk factor	**Chronic diseases (*n =* 163)**						
	No	80 (87.0)	46 (64.8)	1.0		1.0	0.019
	Yes	12 (13.0)	25 (35.2)	3.8 (1.6–8.7)	0.002	3.4 (1.2–9.4)	0.019
	**Alcohol drinking (*n =* 163)**						
	No	89 (96.7)	68 (95.8)	1.0			
	Yes	3 (3.3)	3 (4.2)	1.1 (0.2–5.8)	0.896		
	**Smoking (*n =* 164)**						
	No	87 (93.5)	63 (88.7)	1.0			
	Yes	6 (6.5)	8 (11.3)	1.6 (0.5–4.8)	0.434		
	**Total**	94	71				

Note: 1. *p*-value for group variables by Fisher’s exact test = 0.070 (age), 0.060 (education), 0.015 (marital status) and < 0.001; (religion). *p*-value for a group variable by Chi-square test = 0.175 (financial status); 2. Variables that did not exhibit significant results in the univariate analysis were not included in the multivariate analysis.

## Data Availability

Data available on request due to ethical restrictions.

## Supplementary Materials

The following are available online at https://www.mdpi.com/article/10.3390/ijerph18147269/s1, Supplementary File S1: List of samples in the sex, age and nationality strata, Supplementary File S2: Questions used for assessing anxiety and depression, Supplementary File S3: Multivariate model in three different SDH groups.
