# Supplementary material for: Situation of Self-Reported Anxiety and Depression among Urban Refugees and Asylum Seekers in Thailand, 2019
Source: Int J Environ Res Public Health. 2021 Jul 7;18(14):7269. doi: 10.3390/ijerph18147269 (PMC8307443; doi:10.3390/ijerph18147269)
Supplement: Supplementary file 1 [file ijerph-18-07269-s001.zip › Supplementary files/Supplementary file 1.pdf]

Country	Sex	Age	Total number from BRC list	Total samples required	Total participants attending the survey	Weighted
Afghanistan	male	<15	23	2	1	23.00
Afghanistan	male	≥15	26	2	3	8.67
Afghanistan	female	<15	16	2	1	16.00
Afghanistan	female	≥15	45	2	2	22.50
Cambodia	male	<15	30	2	2	15.00
Cambodia	male	≥15	72	4	4	18.00
Cambodia	female	<15	39	3	1	39.00
Cambodia	female	≥15	57	4	4	14.25
China	male	<15	2	1	2	1.00
China	male	≥15	22	2	1	22.00
China	female	<15	14	2	1	14.00
China	female	≥15	13	2	2	6.50
Iraq	male	<15	12	2	1	12.00
Iraq	male	≥15	32	2	2	16.00
Iraq	female	<15	25	2	1	25.00
Iraq	female	≥15	28	2	3	9.33
Sri Lanka	male	<15	13	2	2	6.50
Sri Lanka	male	≥15	30	2	1	30.00
Sri Lanka	female	<15	31	2	2	15.50
Sri Lanka	female	≥15	21	2	2	10.50
Pakistan	male	<15	243	15	14	17.36
Pakistan	male	≥15	392	25	22	17.82
Pakistan	female	<15	275	18	13	21.15
Pakistan	female	≥15	318	20	23	13.83
Palestine	male	<15	37	2	1	37.00
Palestine	male	≥15	56	4	1	56.00
Palestine	female	<15	33	2	1	33.00
Palestine	female	≥15	52	2	3	17.33
Somali	male	<15	40	2	2	20.00
Somali	male	≥15	72	5	1	72.00
Somali	female	<15	29	2	2	14.50
Somali	female	≥15	20	2	5	4.00
Vietnam	male	<15	183	11	9	20.33
Vietnam	male	≥15	250	16	13	19.23
Vietnam	female	<15	197	13	11	17.91
Vietnam	female	≥15	231	15	12	19.25
Syria	male	<15	9	2	1	9.00
Syria	male	≥15	12	2	3	4.00
Syria	female	<15	11	2	2	5.50
Syria	female	≥15	10	2	1	10.00
