# Supplementary material for: Situation of Self-Reported Anxiety and Depression among Urban Refugees and Asylum Seekers in Thailand, 2019
Source: Int J Environ Res Public Health. 2021 Jul 7;18(14):7269. doi: 10.3390/ijerph18147269 (PMC8307443; doi:10.3390/ijerph18147269)
Supplement: Supplementary file 1 [file ijerph-18-07269-s001.zip › Supplementary files/Supplementary file 2.pdf]

Supplementary file 2: Questions used for assessing anxiety and depression

Anxiety

Currently, regarding <u>your anxiety</u> what do you think about it **most**?

1. ☐ None
2. ☐ Little anxious
3. ☐ Average anxious
4. ☐ Very anxious
5. ☐ Extremely anxious

Depression

In the past 2 weeks, including today, how often do you feel or have these following symptoms?

1. Feeling bored, don't want to do anything

   ☐ None ☐ Sometimes ☐ Often ☐ Everyday

2. Not cheerful, feeling depressed and discouraged

   ☐ None ☐ Sometimes ☐ Often ☐ Everyday

3. Couldn't sleep well OR slept fitfully OR slept too much

   ☐ None ☐ Sometimes ☐ Often ☐ Everyday

4. Feeling tired easily OR exhausted OR fatigue

   ☐ None ☐ Sometimes ☐ Often ☐ Everyday

5. Having problem with eating behaviors, either loss of the appetite OR eating too much

   ☐ None ☐ Sometimes ☐ Often ☐ Everyday

6. Not feeling good with yourself, feeling failed OR feeling that you disappointed your family

   ☐ None ☐ Sometimes ☐ Often ☐ Everyday

7. Lost concentration easily when doing anything e.g. watching TV or doing any jobs that need concentration)

   ☐ None ☐ Sometimes ☐ Often ☐ Everyday

8. Talk slowly OR do things slowly and this can be noticed by other people OR restless OR unable to stand still as usual

   ☐ None ☐ Sometimes ☐ Often ☐ Everyday

9. Think of hurting yourself OR think that everything will be better if you are dead)

   ☐ None ☐ Sometimes ☐ Often ☐ Everyday
