# Supplementary material for: Situation of Self-Reported Anxiety and Depression among Urban Refugees and Asylum Seekers in Thailand, 2019
Source: Int J Environ Res Public Health. 2021 Jul 7;18(14):7269. doi: 10.3390/ijerph18147269 (PMC8307443; doi:10.3390/ijerph18147269)
Supplement: Supplementary file 1 [file ijerph-18-07269-s001.zip › Supplementary files/Supplementary file 3.pdf]

Supplementary file 3: Multivariate model in three different SDH groups

A1: Non-modifiable fators and anxiety

Independent variables	Number of respondents who are not anxious (%)	Number of respondents who are anxious (%)	Adjusted OR (95% CI)	P-value of adjusted OR
Gender				
Female	26 (51.0)	61 (48.4)	1.0	
Male	25 (49.0)	65 (51.6)	1.0 (0.4-2.6)	0.995
Age (years)				
< 15	36 (70.6)	37 (29.4)	1.0	
15-60	14 (27.4)	81 (64.3)	8.4 (3.0-23.4)	<0.001
> 60	1 (2.0)	8 (6.3)	5.7 (0.6-49.6)	0.118
Region				
South East Asia and China	29 (56.9)	40 (31.7)	1.0	
Others	22 (43.1)	86 (68.3)	2.3 (0.5-11.2)	0.289
Religion				
Buddhism	7 (13.7)	8 (6.3)	1.0	
Christ	24 (47.1)	46 (36.5)	3.4 (0.6-20.0)	0.172
Muslim	18 (35.3)	67 (53.2)	3.7 (0.3-38.8)	0.280
Others	2 (3.9)	5 (4.0)	2.6 (0.2-33.7)	0.463
Period of living in Thailand (years) (n=137)				
< 5	19 (54.3)	41 (40.2)	1.0	
≥ 5	16 (45.7)	61 (59.8)	1.6 (0.6-4.1)	0.361
Total	51	126		

A2: Social class fators and anxiety

Independent variables	Number of respondents who are not anxious (%)	Number of respondents who are anxious (%)	Adjusted OR (95% CI)	P-value of adjusted OR
Education				
Up to primary level	40 (78.4)	77 (61.1)	1.0	
Up to secondary level	10 (19.6)	30 (23.8)	0.8 (0.3-2.2)	0.667
Degree or above	1 (2.0)	19 (15.1)	19.9 (2.3-174.7)	0.007
Marital status (n=175)				
Single	41 (82.0)	53 (42.4)	1.0	
Married	8 (16.0)	70 (56.0)	3.7 (1.4-9.6)	0.007
Widow/divorced/separated	1 (2.0)	2 (1.6)	2.5 (0.2-27.4)	0.446
Financial status (n=143)				
Income lower than expense	25 (56.8)	50 (50.5)	1.0	
Income equal to expense	13 (29.6)	30 (30.3)	1.1 (0.4-2.6)	0.900
Income higher than expense	6 (13.6)	19 (19.2)	1.8 (0.5-6.2)	0.372
Total	51	126		

A3: Individual risk factors and anxiety

Independent variables	Number of respondents who are not anxious (%)	Number of respondents who are anxious (%)	Adjusted OR (95% CI)	P-value of adjusted OR
Chronic diseases (n=175)				
No	46 (92.0)	90 (72.0)	1.0	
Yes	4 (8.0)	35 (28.0)	4.6 (1.4-14.7)	0.010
Alcohol drinking (n=174)				
No	50 (98.0)	117 (95.1)	1.0	
Yes	1 (2.0)	6 (4.9)	1.7 (0.2-14.5)	0.615
Smoking (n=175)				
No	49 (96.1)	113 (91.1)	1.0	
Yes	2 (3.9)	11 (8.9)	1.9 (0.3-10.4)	0.473
Total	51	126		

D1: Non-modifiable factors and depression

Independent variables	Number of respondents who are not depressed (%)	Number of respondents who are depressed (%)	Adjusted OR (95% CI)	P-value of adjusted OR
Gender				
Female	45 (47.9)	39 (54.9)	1.0	
Male	49 (52.1)	32 (45.1)	0.7 (0.3-1.6)	0.408
Age (years)				
< 15	46 (48.9)	26 (36.6)	1.0	
15-60	47 (50.0)	40 (56.3)	3.3 (1.3-7.9)	0.009
> 60	1 (1.1)	5 (7.1)	6.9 (1.0-48.8)	0.054
Region				
South East Asia and China	53 (56.4)	13 (18.3)	1.0	
Others	41 (43.6)	58 (81.7)	4.4 (1.0-18.7)	0.043
Religion				
Buddhism	14 (14.9)	1 (1.4)	1.0	
Christ	45 (47.9)	22 (31.0)	4.8 (0.5-45.0)	0.167
Muslim	30 (31.9)	46 (64.8)	7.5 (0.6-100.0)	0.127
Others	5 (5.3)	2 (2.8)	1.5 (0.1-27.9)	0.796
Period of living in Thailand (years) (n=125)				
< 5	31 (49.2)	22 (35.5)	1.0	
≥ 5	32 (50.8)	40 (64.5)	1.0 (0.4-2.5)	0.991
Total	94	71		

D2: Social class factors and depression

Independent variables	Number of respondents who are not depressed (%)	Number of respondents who are depressed (%)	Adjusted OR (95% CI)	P-value of adjusted OR
Education				
Up to primary level	66 (70.2)	44 (62.0)	1.0	
Up to secondary level	24 (25.5)	14 (19.7)	0.5 (0.2-1.5)	0.227
Degree or above	4 (4.3)	13 (18.3)	3.1 (0.8-12.1)	0.107
Marital status (n=163)				
Single	56 (60.9)	34 (47.9)	1.0	
Married	35 (38.0)	34 (47.9)	1.3 (0.5-2.9)	0.587
Widow/divorced/Separated	1 (1.1)	3 (4.2)	4.4 (0.3-56.9)	0.260
Financial status (n=135)				
Income lower than expense	37 (50.7)	35 (56.4)	1.0	
Income equal to expense	19 (26.0)	22 (35.5)	1.0 (0.4-2.4)	0.970
Income higher than expense	17 (22.3)	5 (8.1)	0.4 (0.1-1.2)	0.094
Chronic diseases (n=163)				
No	80 (87.0)	46 (64.8)	1.0	
Yes	12 (13.0)	25 (35.2)	3.8 (1.6-9.1)	0.002
Alcohol drinking (n=163)				
No	89 (96.7)	68 (95.8)	1.0	
Yes	3 (3.3)	3 (4.2)	0.9 (0.2-4.4)	0.934
Smoking (n=164)				
No	87 (93.5)	63 (88.7)	1.0	
Yes	6 (6.5)	8 (11.3)	1.7 (0.4-7.1)	0.434
Total	94	71		

D3: Individual risk factors and depression

Independent variables	Number of respondents who are not depressed (%)	Number of respondents who are depressed (%)	Adjusted OR (95% CI)	P-value of adjusted OR
Chronic diseases (n=163)				
No	80 (87.0)	46 (64.8)	1.0	
Yes	12 (13.0)	25 (35.2)	3.8 (1.6-9.1)	0.002
Alcohol drinking (n=163)				
No	89 (96.7)	68 (95.8)	1.0	
Yes	3 (3.3)	3 (4.2)	0.9 (0.2-4.4)	0.934
Smoking (n=164)				
No	87 (93.5)	63 (88.7)	1.0	
Yes	6 (6.5)	8 (11.3)	1.7 (0.4-7.1)	0.434
Total	94	71		
